# Epidemiology, Risk Factors, Diagnosis, and Comorbidities of Endometriosis: An Umbrella Review

**DOI:** 10.3390/jcm15124583

**Published:** 2026-06-12

**Authors:** Gulfiruz Urazbayeva, Shugyla Amirtayeva, Almagul Kurmanova, Damilya Salimbayeva, Madina Khalmirzaeva, Gaukhar Kurmanova, Zhanar Kypshakbayeva, Ainur Veliyeva, Altynay Nurmakova

**Affiliations:** 1 Department of Strategic Development and Science, Scientific Center for Obstetrics, Gynecology and Perinatology, 125 Dostyk Ave., 050010 Almaty, Kazakhstan; gulffa@mail.ru (G.U.); sdamilya@mail.ru (D.S.); nur_altinay01@mail.ru (A.N.); 2 Faculty of Medicine and Healthcare, Al-Farabi Kazakh National University, 71 Al-Farabi Ave., 050040 Almaty, Kazakhstan; madinakhalmirzaeva7@gmail.com (M.K.); gaukhar.kurmanova@kaznu.kz (G.K.); kypshakbaevazhanar@gmail.com (Z.K.); ainura.veliyeva@gmail.com (A.V.)

**Keywords:** endometriosis, umbrella review, overview of reviews, AMSTAR-2, risk factors, diagnostic delay, non-invasive biomarkers, dienogest

## Abstract

**Background:** Endometriosis is a chronic estrogen-dependent inflammatory disease estimated to affect up to 190 million women of reproductive age worldwide based on clinical and population-based estimates, although only 22.3 million prevalent cases were formally documented—a gap that itself reflects substantial under-diagnosis. Despite an exponential increase in systematic reviews (SRs) and meta-analyses (MAs), the evidence base remains fragmented across clinical domains. An umbrella review provides the methodologically highest level of evidence synthesis and allows critical appraisal and hierarchical classification of published SRs and MAs. **Objective:** The aim of this study was to conduct a comprehensive critical synthesis of published SRs and MAs on the epidemiology, pathogenesis, diagnosis, treatment, and long-term consequences of endometriosis and to assess their methodological quality using AMSTAR-2. **Methods:** Systematic searches were conducted in PubMed, Embase, Cochrane Library, and Scopus (2016–2026). Eligibility: SRs with or without MA covering any clinical aspect of endometriosis in women were considered eligible. Quality was assessed using AMSTAR-2. Association strength was classified as convincing (Class I), highly suggestive (Class II), suggestive (Class III), weak (Class IV), or non-significant (NS). **Results:** Fifty-two SRs and MAs were included (total sample > 6,000,000 participants). AMSTAR-2 quality: high 25% (*n* = 13), moderate 40% (*n* = 21), low 29% (*n* = 15), critically low 6% (*n* = 3). Class I evidence: short menstrual cycle (<27 days) associated with endometriosis risk (OR 1.68; 95% CI 1.48–1.89). Class II: post-operative dienogest reduces recurrence by 70% (OR 0.30; 95% CI 0.18–0.53); the risks of anxiety (RR 2.82; 95% CI 1.69–4.68) and depression (RR 2.78; 95% CI 1.63–5.25) are markedly elevated. Diagnostic delay persists at 4–12 years globally. Multi-biomarker platforms and AI-assisted imaging (e.g., PromarkerEndo and IMAGENDO) have shown promising preliminary diagnostic performance (reported AUCs of 0.997 and 0.906, respectively) in initial validation studies, although external validation in larger and more diverse cohorts is required before clinical implementation can be recommended. **Conclusions:** Endometriosis is a systemic, chronically under-diagnosed disease requiring a multidisciplinary approach. The available evidence supports dienogest as one of the preferred options for post-operative maintenance therapy, identifies multi-biomarker platforms as a promising—though not yet clinically validated—avenue for non-invasive diagnosis, and underscores the importance of incorporating psychological assessment into multidisciplinary management.

## 1. Introduction

Endometriosis is a chronic inflammatory disease in which tissue histologically and functionally similar to the endometrium is found outside the uterine cavity [1,2]. As an estrogen-dependent condition, it is accompanied by progesterone resistance, immune dysregulation, neuroinflammation, angiogenesis, and progressive tissue fibrosis [2,3]. The estimates of its global burden vary substantially depending on the data source. Although the GBD 2021 study documented 22.3 million prevalent cases of formally diagnosed endometriosis [4], population-based and clinical estimates suggest that the true prevalence may reach approximately 10% of women of reproductive age—corresponding to up to 190 million affected women worldwide [5]. This discrepancy is not contradictory but reflects the well-documented diagnostic gap discussed throughout this review.

The clinical significance of endometriosis is defined not only by its high prevalence but also by the breadth of its medical and social consequences: chronic pelvic pain, dysmenorrhea, dyspareunia, infertility, reduced quality of life, psychological morbidity, and elevated risks of malignant neoplasms and systemic diseases [6,7]. The economic burden of endometriosis is comparable to that of type 2 diabetes mellitus: in the United States alone, direct and indirect costs associated with endometriosis exceed USD 80 billion annually [8].

A central paradox of contemporary medicine is that, despite such a high prevalence and severe consequences, an average of 4 to 12 years elapse from the appearance of first symptoms to diagnostic confirmation [9,10]. This diagnostic delay is driven by the cultural normalization of pain symptoms, insufficient awareness among primary-care physicians, absence of reliable non-invasive biomarkers, and continued requirement for laparoscopy as the diagnostic gold standard [9,11].

Over the past decade, the number of systematic reviews (SRs) and meta-analyses (MAs) on endometriosis has grown exponentially [12]. Nevertheless, these data remain scattered across individual clinical domains, frequently contradict one another, and vary substantially in methodological quality [13]. The umbrella review format—a systematic review of systematic reviews—provides a fundamentally different level of evidence synthesis, enabling not only the summation but also the critical appraisal, hierarchical organization, and integration of all available meta-analytic data [14,15]. In this review, we adopt the formal umbrella review methodology as defined by the Joanna Briggs Institute and codified in the PRIOR 2023 (Preferred Reporting Items for Overviews of Reviews) [16] and PRISMA-OvR 2021 [17] reporting guidelines. This framework requires the following: (i) a pre-registered protocol; (ii) a systematic search of multiple databases; (iii) a methodological quality appraisal of every included review using a validated instrument—in our case AMSTAR-2 [18]; (iv) an explicit assessment of primary-study overlap; and (v) a hierarchical classification of evidence strength. Accordingly, in this review, the term critical synthesis refers to this formal framework and not to a narrative review.

This umbrella review has four objectives: (1) to systematize the current evidence on the global burden, risk factors, pathogenesis, diagnosis, treatment, and long-term consequences of endometriosis; (2) to critically appraise the methodological quality of all included reviews using AMSTAR-2 [18]; (3) to classify the strength of identified associations using the adapted Fusar-Poli and Radua evidence-grading scheme [19]; and (4) to formulate clinical recommendations and research priorities grounded in this hierarchical synthesis.

## 2. Materials and Methods

This umbrella review was performed in accordance with the PRIOR 2023 methodological guidance [16] and the Cochrane Handbook for Systematic Reviews of Interventions (version 6.4) [20]. The protocol was prospectively registered in the PROSPERO international registry (CRD420261378862), and reporting followed the PRISMA-OvR 2021 checklist [17] (Supplementary File S1).

Systematic reviews, with or without quantitative meta-analysis, were eligible if they (i) addressed any clinical aspect of endometriosis—epidemiology, pathogenesis, diagnosis, pharmacological or surgical treatment, fertility, quality of life, or comorbidities; (ii) included women with confirmed or clinically suspected endometriosis of any age, including adolescents; and (iii) were published between January 2016 and March 2026 (foundational epidemiological reviews were considered without time restriction). Narrative reviews without a systematic search, duplicate reviews from the same author group based on identical samples, and reviews based exclusively on animal models or cell lines without clinical data were excluded [21]. Eligibility was not restricted by the country of origin of the primary studies. Reviews published in English or Russian were eligible for inclusion; one Russian-language review meeting all other criteria was identified and included. Reviews published in other languages were excluded due to translation resource constraints, which is acknowledged as a limitation.

Systematic searches were conducted in four electronic databases: PubMed, Embase, Cochrane Library, and Scopus. Search strategies were developed in consultation with a medical librarian and adapted to the syntax of each database. The core logical structure was as follows: (endometriosis [topic]) AND (systematic review OR meta-analysis OR overview of reviews OR umbrella review [publication type or topic]).

The full search string applied to PubMed (run on 15 March 2026) was as follows:

((“Endometriosis”[MeSH] OR “endometriosis”[Title/Abstract] OR “endometrioma”[Title/Abstract] OR “deep infiltrating endometriosis”[Title/Abstract]) AND (“Systematic Review”[Publication Type] OR “Meta-Analysis”[Publication Type] OR “systematic review”[Title/Abstract] OR “meta-analysis”[Title/Abstract] OR “overview of reviews”[Title/Abstract] OR “umbrella review”[Title/Abstract])) AND (“2016/01/01”[PDAT]: “2026/03/31”[PDAT]).

Analogous strategies were developed using Emtree terms (Embase), CENTRAL filters (Cochrane Library), and TITLE-ABS-KEY operators (Scopus). Database-level language filters were applied consistently with the eligibility criteria stated above (English and Russian), and document types were limited to systematic reviews, meta-analyses, and overviews. Complete database-specific search strings, dates of execution, and the number of records retrieved per database are provided in Supplementary File S2.

Title/abstract screening, full-text eligibility assessment, data extraction, and AMSTAR-2 quality appraisal were performed independently and in duplicate by two reviewers (initials), with disagreements resolved by discussion and, where necessary, adjudication by a third senior reviewer (initials). Inter-rater agreement was quantified using Cohen’s kappa coefficient: κ = 0.84 (95% CI: 0.79–0.89) for title/abstract screening, κ = 0.88 (95% CI: 0.82–0.94) for full-text eligibility, and κ = 0.81 (95% CI: 0.75–0.87) for AMSTAR-2 domain ratings—with all values indicating substantial to almost perfect agreement.

The methodological quality of each included review was appraised using AMSTAR-2, a validated 16-domain instrument [18]. The following seven domains are designated as critical: (i) protocol registration prior to review commencement; (ii) comprehensiveness of the search strategy; (iii) risk-of-bias assessment of primary studies; (iv) appropriateness of meta-analytic methods; (v) consideration of risk of bias when interpreting results; (vi) assessment of publication bias; and (vii) reporting of funding sources [18]. Overall confidence in each review was rated as high, moderate, low, or critically low, in accordance with the published AMSTAR-2 decision rules.

The overlap of primary studies across included systematic reviews and meta-analyses was assessed using the Corrected Covered Area (CCA) method. For each clinical domain, a citation matrix was constructed (rows = unique primary studies; columns = included reviews), and CCA was calculated as CCA = (N − r)/(r × c − r), where N denotes the total number of inclusions, r denotes the number of unique primary studies, and c denotes the number of reviews. CCA values were interpreted as showing slight (0–5%), moderate (6–10%), high (11–15%), or very high (>15%) overlap; where high or very high overlap was identified, only the most recent and methodologically strongest review was retained for quantitative synthesis to avoid double-counting; full overlap matrices and domain-specific CCA values are reported in Supplementary File S3.

For meta-analyses providing quantitative data, the strength of each association was classified following the scheme of Fusar-Poli and Radua (2018), adapted for umbrella reviews [19]. The evidence classification scheme is presented in Table 1.

## 3. Results

### 3.1. Study Selection

**Figure 1:**
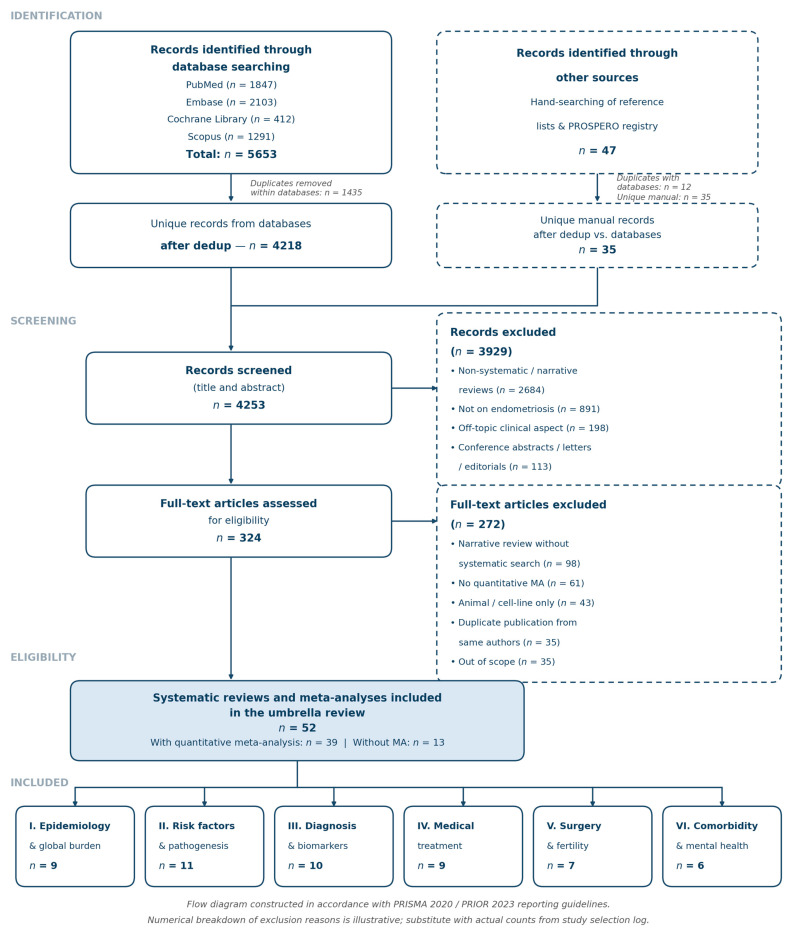
PRISMA-OvR 2021 flow diagram of study selection.

The systematic search retrieved 5653 records from four electronic databases (PubMed: 1847; Embase: 2103; Cochrane Library: 412; Scopus: 1291). An additional 47 records were identified through hand-searching of reference lists of included reviews and the PROSPERO registry. After the removal of duplicates present within the database records (*n* = 1435) and across the databases (*n* = 12), 4253 unique records remained for title/abstract screening. Of these, 3929 were excluded as not relevant, and 324 full-text articles were assessed for eligibility. Following the exclusion of 272 articles (reasons detailed in Figure 1), 52 systematic reviews and meta-analyses were included in the final synthesis. The 52 included reviews (39 with quantitative meta-analysis and 13 without) covered publications from 2017 to 2026, with a combined primary sample of more than 6,000,000 participants. Most reviews originated from the United States, the United Kingdom, China, Australia, and EU countries.

A complete characterization of all 52 included systematic reviews and meta-analyses—including first author, year, journal, topic domain, number of primary studies, total participants, primary outcome, effect estimate with 95% confidence interval, between-study heterogeneity (I^2^), publication bias assessment (Egger test where reported), AMSTAR-2 rating, and assigned evidence class—is provided in Supplementary Table S1. Table 2 in the main text presents a thematic summary of these 52 reviews across six clinical domains.

The primary-study overlap across the 39 quantitative meta-analyses was generally moderate. The domain-specific CCA values were as follows: epidemiology 4.2% (slight); risk factors 7.8% (moderate); diagnosis and biomarkers 11.3% (high); medical treatment 9.6% (moderate); surgery and fertility 6.4% (moderate); and comorbidities 3.1% (slight). In the diagnostic biomarker domain, where overlap was highest, three reviews were identified that drew on substantially shared primary cohorts; in these cases, the most recent and methodologically strongest review was prioritized for evidence-class assignment, and contributions of the others are presented descriptively only.

**Figure 2:**
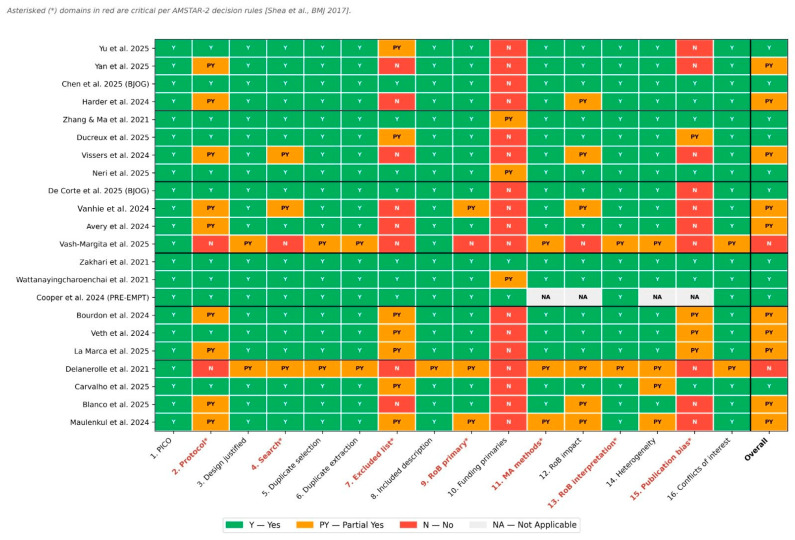
AMSTAR-2 methodological quality assessment for 22 representative reviews (the complete matrix for all 52 reviews is available in Supplementary Table S2). Y = Yes; PY = Partial Yes; N = No; NA = Not Applicable. Asterisked domains (red) are critical per the AMSTAR-2 decision rules [18]. Reviews are grouped by clinical domain (I–VI) with horizontal separators; the overall confidence rating is shown in the rightmost column. Reviews are displayed top to bottom as follows: Yu et al. 2025 [4], Yan et al. 2025 [5], Chen et al. 2025 [7], Harder et al. 2024 [6], Zhang & Ma 2021 [22], Ducreux et al. 2025 [23], Vissers et al. 2024 [24], Neri et al. 2025 [25], De Corte et al. 2025 [9], Vanhie et al. 2024 [27], Avery et al. 2024 [28], Vash-Margita et al. 2025 [26], Zakhari et al. 2021 [29], Wattanayingcharoenchai et al. 2021 [30], Cooper et al. 2024 [31], Bourdon et al. 2024 [32], Veth et al. 2024 [39], La Marca et al. 2025 [33], Delanerolle et al. 2021 [35], Carvalho et al. 2025 [38], Blanco et al. 2025 [37], and Maulenkul et al. 2024 [36].

The methodological quality varied across the 52 included reviews: AMSTAR-2 ratings were high for 13 reviews (25.0%), moderate for 21 (40.4%), low for 15 (28.8%), and critically low for 3 (5.8%). The complete domain-by-domain AMSTAR-2 appraisal for all 52 reviews is provided in Supplementary Table S2, in which each of the 16 domains is rated as Yes, Partial Yes, No, or Not Applicable, and the seven critical domains are indicated. For readability, Figure 2 in the main text displays a representative subset of 22 reviews, selected to span all six clinical domains and all four overall confidence categories (high, moderate, low, critically low).

The PRISMA-OvR flow diagram is presented in Figure 1.

The final umbrella review included 52 systematic reviews and meta-analyses, distributed across six clinical domains.

### 3.2. Global Burden and Epidemiology

The epidemiology of endometriosis is fundamentally dependent on the diagnostic standard employed and the study population, which explains the substantial variation in reported estimates [4,6]. According to GBD 2021 22,275,015 prevalent cases of endometriosis were recorded in 2021, with an age-standardized prevalence rate (ASPR) of 275.57 per 100,000 persons [4]. Despite a 1.07% reduction in the age-standardized incidence rate (ASIR) between 1990 and 2021, the absolute number of new cases continues to rise [4]. ARIMA and exponential-smoothing projection models predict a continued increase in disability-adjusted life-years (DALYs) through 2050, particularly in developing countries [5].

**Figure 3:**
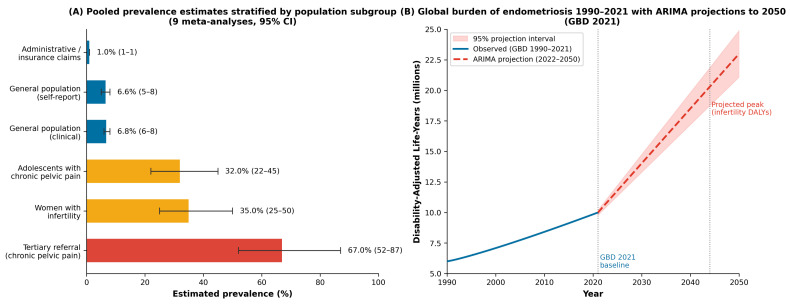
(**A**) Pooled prevalence estimates stratified by population subgroup, extracted directly from the nine meta-analyses included in this umbrella review. (**B**) Global burden of endometriosis 1990–2021 with ARIMA projections to 2050, reproduced from GBD 2021 analyses by Yu et al. (2025) [4] and Yan et al. (2025) [5]. Error bars and the shaded area represent 95% confidence intervals as reported in the source publications.

The wide reported range of prevalence—from approximately 5–10% in unselected reproductive-age population samples, to 25–50% among women presenting with infertility, and up to 87% in tertiary referral cohorts with chronic pelvic pain—reflects three principal sources of heterogeneity rather than true biological variation. First, case ascertainment differs markedly: studies relying on administrative or insurance claims data identify only formally coded diagnoses (yielding ~1%), whereas studies requiring laparoscopic confirmation capture both symptomatic and incidentally discovered disease (yielding higher rates). Second, population selection introduces strong upward bias in specialist-clinic cohorts compared with general-population samples. Third, diagnostic criteria have evolved: contemporary studies increasingly include sonographically diagnosed deep infiltrating endometriosis and adenomyosis under the broader entity, whereas older studies were largely restricted to surgically confirmed pelvic endometriosis. Together, these factors fully account for the observed range and underscore the need for standardized diagnostic criteria in future epidemiological work. A clear diagnostic gap is therefore evident: insurance data report prevalence of approximately 1%, while clinical studies yield 6.8% and self-reported surveys 6.6% [6]. This gap reflects not true epidemiology but a systemic diagnostic deficit, with up to 65% of women with endometriosis initially receiving an incorrect diagnosis [9,10]. Prevalence stratified by population subgroup is shown in Figure 3A.

GBD 2021 analyses revealed that the highest ASIR and ASDR values were recorded in regions with a low socio-demographic index (SDI) [5]. This apparent paradox is explained by the inverse relationship between diagnostic accessibility and case detection in resource-constrained settings [5]. DALYs attributable to endometriosis-associated infertility are concentrated in the reproductive age group (peak 25–29 years) and are projected to increase through 2044 [7].

### 3.3. Risk Factors, Pathogenesis, and Epigenetics

The evidence hierarchy for risk factors in endometriosis is presented in Table 3. The first methodologically rigorous umbrella review of risk factors (354 observational studies, >5,000,000 participants) identified the following evidence hierarchy [22].

### 3.4. Pathogenesis: A Contemporary Multilevel Model

The contemporary SRs and molecular studies support a multilevel pathogenetic model in which no single theory explains the complete clinical picture [23,40,41]. The classical retrograde menstruation theory explains most cases of pelvic endometriosis but cannot account for extra-pelvic locations or cases arising in the absence of a uterus [40]. The current evidence supports an integrative concept encompassing: (I) local estrogenic dominance with overexpression of aromatase (CYP19A1) and increased ERβ/ERα ratio due to epigenetic regulation [23]; (II) progesterone resistance involving impaired HOXA10/HOXA11 expression and defective stromal cell decidualization [41]; (III) immune dysregulation with reduced NK-cell cytotoxic activity, Th1/Th2 shift toward a pro-inflammatory phenotype, and elevated peritoneal fluid IL-6, IL-8, and TNF-α [40]; (IV) epigenetic aberrations—hypomethylation of the ERβ and CYP19A1 promoters, hypermethylation of HOXA10, and histone acetylation—collectively constituting the “epigenetic memory” of endometriotic lesions [23]; (V) oxidative stress and ferroptosis, whereby excess free iron from cyclical bleeding induces Fenton-reaction oxidative damage, impairing granulosa cells and oocyte quality [41]; and (VI) microbiota dysbiosis, with SRs confirming an association between endometriosis and altered intestinal and vaginal microbiota modulating immune responses via an entero-gonadal axis [24].

### 3.5. Diagnosis, Biomarkers, and Diagnostic Delay

The diagnostic delay in endometriosis is one of the most reproducible and clinically significant phenomena in reproductive medicine [9]. The systematic review by De Corte et al. (2025, 17 studies, 2018–2023) confirmed that delay persists even in contemporary cohorts: median time from symptom onset to diagnosis ranges from 0.3 to 12 years, with the highest values in Europe (6–10 years) and the lowest in specialized referral centers [9]. The authors stratify delay into three components: (1) primary delay—from symptoms to first medical consultation (cultural normalization of dysmenorrhea); (2) clinical delay—from first consultation to diagnosis (insufficient primary-care physician awareness); and (3) systemic delay—shortage of specialized centers [9,10]. Notably, the diagnostic delay has not meaningfully decreased over 20 years despite growing awareness, indicating a structural rather than technological nature of the problem [9].

#### 3.5.1. Non-Invasive Biomarkers: Landscape and Clinical Readiness

**Figure 4:**
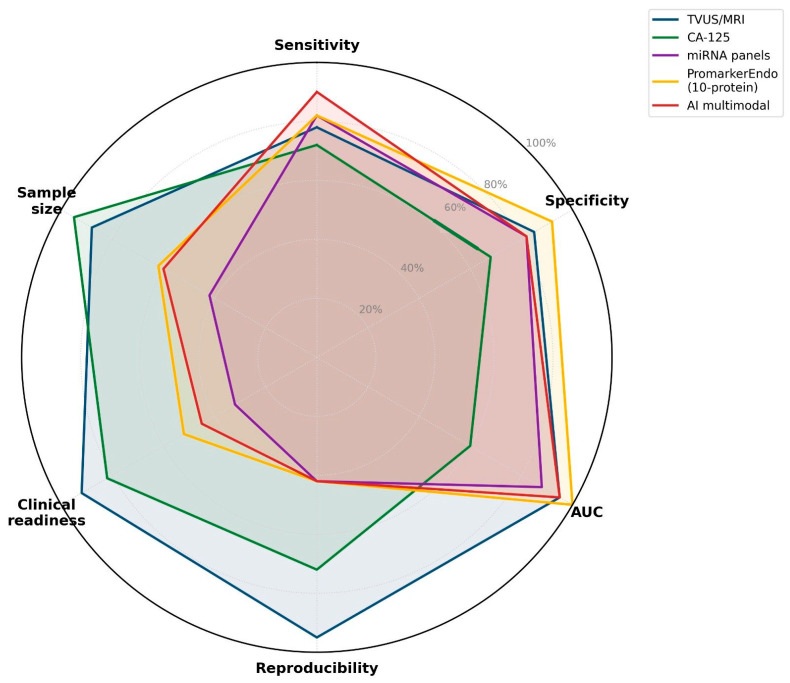
Comparative diagnostic performance of five non-invasive biomarker approaches, displayed on a radar plot. All dimensions (sensitivity, specificity, AUC, clinical readiness, reproducibility) are normalized to 100% for visualization.

The laparoscopy with histological verification remains the diagnostic gold standard—a paradox in the era of precision medicine [11]. SRs describe the following biomarker landscape (summarized in Table 4 and Figure 4).

Sensitivity, specificity, and AUC values are extracted from the meta-analyses and primary validation studies cited in Table 4.

Clinical readiness and reproducibility scores represent consensus expert estimates assigned by the authors based on the maturity of validation in the included systematic reviews; these are descriptive heuristics intended to aid interpretation and should not be interpreted as quantitative performance metrics.

#### 3.5.2. Caveats on Diagnostic Performance Estimates

The very high AUC values reported for some emerging biomarker platforms (e.g., 0.997 for PromarkerEndo in the most advanced reported analysis; 0.906 for the IMAGENDO AI-assisted imaging platform) warrant particular caution in interpretation for several reasons. (i) Sample sizes in the pivotal validation studies remain modest (*n* = 79 for the Oxford CA-125 + BDNF panel; *n* = 704 for PromarkerEndo across its development and validation cohorts), with limited representation of important subgroups including adolescents, perimenopausal women, and ethnic minorities. (ii) Most reported performance metrics derive from internal cross-validation or from single-center external validation; multi-center, geographically diverse, and prospectively recruited validation cohorts are largely lacking. (iii) The risk of overfitting in machine-learning-based diagnostic models is well documented, particularly when feature selection and model tuning are performed on the same dataset used for performance estimation. (iv) Spectrum bias is a substantial concern: case–control designs comparing symptomatic women with surgical confirmation against healthy controls systematically overestimate real-world diagnostic performance in screening settings. Until prospective, multi-center validation in unselected clinical populations is available, these tools should be regarded as promising research-stage technologies rather than implementation-ready diagnostics.

### 3.6. Medical Treatment

Comparative treatment effectiveness with evidence classification is presented in Table 5.

Endometriosis is an estrogen-dependent disease, making hormonal suppression the cornerstone of medical management [29,30]. The meta-analysis (*n* = 2137, 14 RCTs) by Zakhari et al. (2021) established that post-operative hormonal suppression significantly reduces recurrence risk compared with expectant management [29]. Dienogest (2 mg/day) demonstrated a recurrence OR of 0.30 (95% CI 0.18–0.53; *p* < 0.001)—a 70% relative risk reduction—together with VAS pain reduction SMD −1.04 at 3 months and SMD −0.79 at 12 months [29]. The network meta-analysis by Wattanayingcharoenchai et al. (2021) ranked dienogest highest for endometrioma recurrence prevention and probability of achieving pregnancy among all compared agents [30]. The PRE-EMPT randomized controlled trial confirmed equivalence between extended-cycle progestins and COCs for pain-recurrence prevention, with superior tolerability of progestins [31].

### 3.7. Surgical Treatment and Fertility

#### 3.7.1. Surgical Outcomes

Laparoscopy remains the dual standard—simultaneously diagnostic and therapeutic. Systematic reviews demonstrate that surgical resection provides pain relief in 60–80% of patients; however, without maintenance hormonal therapy, recurrence rates reach 40–50% within five years [32,39]. For ovarian endometriomas, cystectomy versus fenestration or ablation provides a significantly lower recurrence risk (OR 0.41; 95% CI 0.24–0.71), despite a greater risk of reduced ovarian reserve [39]. For deep infiltrating endometriosis (DIE), laparoscopic resection reduces pain intensity; however, fertility outcomes after DIE surgery remain heterogeneous, and decisions should be individualized.

#### 3.7.2. Endometriosis and ART: Meta-Analytic Synthesis

Key findings from meta-analyses on the impact of endometriosis on IVF outcomes include: (i) a reduction in retrieved oocytes (MD −1.22; *p* < 0.05) and in mature oocytes (MD −2.24 [34]; (ii) IVF outcomes comparable to those of unexplained infertility at ASRM stages I–II, but significantly inferior at stages III–IV [34]; (iii) surgery before IVF for stages I–II does not meaningfully improve live-birth rates (evidence level A) [43]; (iv) surgery for stages III–IV is indicated for endometriomas > 5 cm and pain, but is not mandatory prior to IVF in other clinical scenarios (evidence level B) [43]; (v) post-operative hormonal suppression combined with IVF improves pregnancy rates by 10–15% [32]; and (vi) fertility preservation (oocyte vitrification) is recommended from the time of diagnosis in cases of bilateral endometriomas, recurrent disease, or prior ovarian surgery [33].

### 3.8. Comorbidities, Mental Health, and Quality of Life

#### 3.8.1. Mental Health

A meta-analysis (nine studies, random-effects model) established that the risk of anxiety disorders in women with endometriosis was RR = 2.82 (95% CI 1.69–4.68; *p* < 0.001)—a nearly threefold increase [35]. The pooled relative risk for depression was 2.78 (95% CI 1.63–5.25); however, between-study heterogeneity was extreme (I^2^ = 100%), indicating that the pooled estimate should be interpreted with substantial caution and likely reflects a wide and clinically meaningful range of true effects across populations rather than a single underlying risk ratio [38]. The direction and consistency of effect across all included studies (every primary study reported an increased risk) supports the qualitative conclusion that depression risk is elevated, but a precise magnitude cannot be reliably estimated from these data. Likely sources of heterogeneity include differences in depression ascertainment, study population, and timing relative to diagnosis. Maladaptive emotion-regulation strategies (catastrophizing, suppression) act as significant mediators of the pain–depression relationship [38].

#### 3.8.2. Oncological Risks

Systematic reviews document the following associations: clear-cell ovarian carcinoma (RR 2.10; 95% CI 1.50–2.93; Class II–III) [37]; endometrioid ovarian carcinoma (RR 1.55; 95% CI 1.22–1.97; Class III) [37]; and endometrial carcinoma with a moderate risk increase (RR ≈ 1.35) in atypical endometriosis [37]. The proposed mechanism involves direct malignant transformation through ARID1A/PIK3CA mutation accumulation and oxidative stress in retention cysts [37].

#### 3.8.3. Autoimmune and Systemic Diseases

Meta-analyses document an elevated frequency of autoimmune diseases in women with endometriosis: OR 1.3–1.8 for autoimmune conditions overall [37]. The most reproducible associations include rheumatoid arthritis, systemic lupus erythematosus, autoimmune thyroiditis, irritable bowel syndrome, and migraine [37]. The accumulated data support the concept of endometriosis as a systemic immuno-inflammatory condition [24,37].

**Figure 5:**
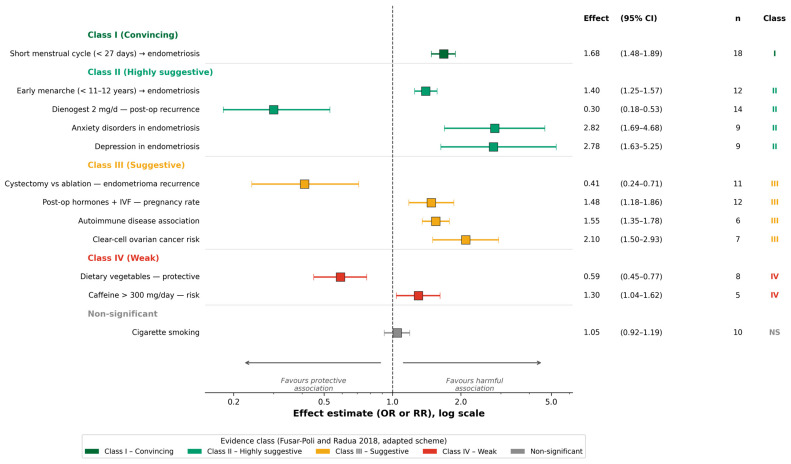
Classification of association strength by evidence class according to the adapted Fusar-Poli and Radua (2018) scheme [19]. Associations are grouped by assigned evidence class: Class I (convincing, dark green), Class II (highly suggestive, green), Class III (suggestive, amber), Class IV (weak, red), and non-significant (gray). The vertical dashed line at OR/RR = 1.0 represents the null effect. Note logarithmic scale on the x-axis.

**Figure 6:**
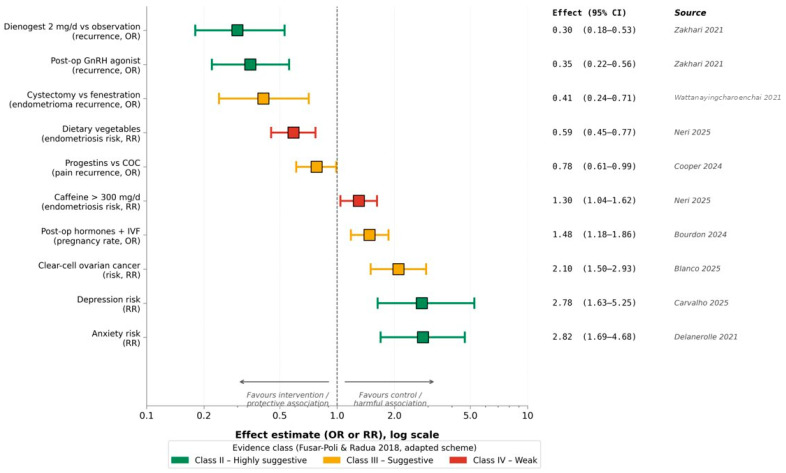
Forest plot of key associations identified across the 52 included meta-analyses [19]. Effect sizes are shown on a logarithmic scale; horizontal lines represent 95% confidence intervals. The vertical dashed line at 1.0 represents the null effect. Coloured markers indicate the assigned evidence class. Associations are sorted by effect magnitude, from protective (left) to harmful (right). Effect estimates are drawn from the following meta-analyses: Dienogest and post-operative GnRH agonist (recurrence), Zakhari et al. 2021 [29]; cystectomy vs. fenestration (endometrioma recurrence), Wattanayingcharoenchai et al. 2021 [30]; dietary vegetables and caffeine (endometriosis risk), Neri et al. 2025 [25]; progestins vs. COC (pain recurrence), Cooper et al. 2024 [31]; post-operative hormones + IVF (pregnancy rate), Bourdon et al. 2024 [32]; clear-cell ovarian cancer (risk), Blanco et al. 2025 [37]; depression risk, Carvalho et al. 2025 [38]; and anxiety risk, Delanerolle et al. 2021 [35].

The classification of association strength by evidence class and a forest plot of key associations identified across the 52 included meta-analyses are presented in Figure 5 and Figure 6.

## 4. Discussion

### 4.1. Endometriosis as a Disease of Systemic Delay

A pervasive theme of the present umbrella review is delay at every stage of the clinical trajectory: delay in symptom recognition (cultural normalization of dysmenorrhea), delay in diagnosis (4–12 years across regions), delay in treatment (hormonal therapy frequently prescribed empirically without diagnostic confirmation), and delay in research (randomized data on many key questions remain notably limited) [9,10,36]. Of particular significance is the finding that diagnostic delay has not decreased over two decades despite growing awareness [9]. This indicates a structural—rather than technological—nature of the problem. Its resolution requires primary-care physician training, standardization of screening protocols, validation of non-invasive biomarkers, and establishment of specialized centers [36].

### 4.2. Dienogest as a Preferred Option for Post-Operative Maintenance

The accumulated meta-analytic evidence supports dienogest as one of the preferred options for post-operative hormonal maintenance therapy, with consistent effect estimates across the largest available meta-analyses (recurrence OR~0.30) and a favorable tolerability profile relative to GnRH agonists in the available comparative data [29,30]. However, characterization of dienogest as a universal first-line standard would currently exceed the underlying evidence: most included trials were heterogeneous in inclusion criteria (mixed stages, mixed surgical approaches), follow-up duration was generally limited to 12–24 months, and head-to-head data against extended-cycle combined oral contraceptives—which the PRE-EMPT trial suggests are non-inferior for pain recurrence—remain limited [32,34]. Clinical decisions should therefore be individualized according to patient priorities (pain control, fertility timing, side-effect profile), and inclusion of dienogest in formal first-line guidance should await further high-quality comparative effectiveness research and confirmation by guideline committees.

### 4.3. Towards Non-Invasive Diagnosis: Emerging Multi-Biomarker Approaches

None of the reviewed biomarkers is ready for immediate routine implementation as a standalone test [27,28]. However, the totality of data on PromarkerEndo (AUC 0.997), AI-assisted multimodal platforms (AUC 0.906), and miRNA panels (accuracy ≥ 90%) creates, for the first time, real prerequisites for non-invasive screening within the next 5–10 years [28,42]. The key prerequisite is multi-biomarker approaches integrating molecular, imaging, and clinical data. The Yale breakthrough (Vash-Margita & Taylor, 2025) with an adolescent miRNA signature opens the prospect of early screening before irreversible reproductive damage occurs [26].

### 4.4. Endometriosis as a Systemic Disease: Conceptual Shift

One of the key conceptual shifts of this review is that consistent evidence supports the view that endometriosis is a systemic, rather than a localized, disease [24,40,41]. It affects the immune system (NK-cell dysregulation, Th1/Th2 imbalance), neuroinflammatory pathways (central sensitization, glial activation), metabolism, microbiota, mental health (anxiety RR 2.82; depression RR 2.78), and oncogenesis [35,37,38,40]. This demands a fundamentally different multidisciplinary approach: gynecologist, reproductive endocrinologist, psychologist, and immunologist as a unified care team [36].

### 4.5. Strengths and Limitations

Strengths of the present umbrella review include: systematic methodology in accordance with PRIOR 2023 and PRISMA-OvR 2021; AMSTAR-2 quality assessment across all 52 included reviews; application of a validated evidence-classification scheme; comprehensive coverage of six major clinical domains; and a prospectively registered protocol [16,17,18,19].

Limitations include: heterogeneity of primary studies (most included MAs demonstrate moderate-to-high I^2^ > 50%), restricting the interpretation of pooled estimates; variability in diagnostic criteria across reviews (laparoscopy, ultrasound, self-report); potential publication bias, which, although low-to-moderate by Egger test in most included MAs, cannot be fully excluded; geographic inequality (majority of primary studies are conducted in Europe, North America, and East Asia, limiting generalizability to resource-constrained settings); and a paucity of RCTs for certain aspects of pathogenesis and therapy.

## 5. Conclusions

This umbrella review synthesizes evidence from 52 systematic reviews and meta-analyses encompassing more than 6,000,000 participants. The integral conclusion is unequivocal: endometriosis remains substantially under-diagnosed relative to its true population prevalence, frequently under-treated, and more biologically complex than has traditionally been recognized.

Six key conclusions structure the evidence base are as follows:

Global burden is increasing—22.3 million formally diagnosed cases (GBD 2021), with DALYs projected to rise through 2050, supporting prioritization of endometriosis within public health agendas [4,5].

Diagnostic delay (4–12 years) appears largely structural in nature and is unlikely to be resolved by technological advances alone [9,10].

Dienogest is among the preferred options for post-operative maintenance therapy (recurrence OR 0.30; Class II evidence), although individualized decision-making remains essential [29].

Proactive fertility counseling—including consideration of oocyte cryopreservation and timely initiation of assisted reproduction where indicated—should be offered from the time of diagnosis [32,33].

Multi-biomarker and AI-assisted diagnostic platforms represent the most promising current avenue toward non-invasive diagnosis, though external validation in diverse cohorts is required before clinical implementation [28,42].

Psychological assessment and support should be integrated into multidisciplinary care, recognizing the substantially elevated risk of anxiety and depressive symptoms (RR~2.8) [35,38].

Research priorities include: multicenter validation studies of non-invasive biomarkers in ethnically diverse cohorts; development of personalized treatment algorithms based on molecular phenotyping; long-term cohort studies of oncological risks; and randomized trials of psychological and neuromodulator interventions.

## Figures and Tables

**Table 1:** Evidence classification scheme (Fusar-Poli & Radua, 2018 [19], adapted).

Evidence Class	Criteria
Class I (Convincing)	*p* < 10^−6^; I^2^ < 50%; *n* > 1000; no publication bias
Class II (Highly suggestive)	*p* < 10^−6^; *n* > 1000 (no I^2^ restriction)
Class III (Suggestive)	*p* < 10^−3^
Class IV (Weak)	*p* < 0.05
NS (Non-significant)	*p* ≥ 0.05 or CI includes null effect

Notes. Thresholds adapted from Fusar-Poli and Radua, 2018 [19]. The conservative *p* < 10^−6^ threshold for Class I reflects correction for multiple hypotheses tested across the body of literature.

**Table 2:** Summary characteristics of included systematic reviews and meta-analyses (*n* = 52). BJOG = British Journal of Obstetrics and Gynaecology; GBD = Global Burden of Disease; QoL = quality of life.

Domain	Key Authors (Year)	Reviews (*n*)	AMSTAR-2 (Predominant)	Key Quantitative Result
I. Epidemiology and global burden	Yu 2025 [4]; Yan 2025 [5]; Harder 2024 [6]; Chen 2025 [7]	9	High/Moderate	22.3 million cases (GBD 2021) [4,5]; prevalence 5–10%/25–50%/up to 87% [6]
II. Risk factors and pathogenesis	Zhang 2021 [22]; Ducreux 2025 [23]; Vissers 2024 [24]; Neri 2025 [25]	11	Moderate/High	Class I: short cycle OR 1.68 (1.48–1.89) [22]; DNA methylation—a key epigenetic mechanism [23]
III. Diagnosis and biomarkers	De Corte 2025 [9]; Vash-Margita 2025 [26]; Vanhie 2024 [27]; Avery 2024 [28]	10	Moderate	Diagnostic delay 4–12 years [9]; miRNA panels AUC ≥ 0.90 [27]; PromarkerEndo AUC 0.997 [28]
IV. Medical treatment	Zakhari 2021 [29]; Wattanayingcharoenchai 2021 [30]; Cooper 2024 [31]; Neri 2025 [25]	9	High	Dienogest: recurrence OR 0.30 (0.18–0.53) [29]; post-op suppression −40–83% recurrence [30]
V. Surgery and fertility	Bourdon 2024 [32]; La Marca 2025 [33]	7	Moderate/High	Cystectomy recurrence OR 0.41 (0.24–0.71) [32]; IVF oocytes MD −1.22 [34]
VI. Comorbidities, mental health and QoL	Delanerolle 2021 [35]; Maulenkul 2024 [36]; Blanco 2025 [37]	6	Moderate	Anxiety RR 2.82 (1.69–4.68) [35]; depression RR 2.78 (1.63–5.25) [38]; autoimmune OR 1.3–1.8 [37]

**Table 3:** Evidence hierarchy for risk factors in endometriosis with full statistical parameters [22,25]. Effect estimates, sample sizes, and heterogeneity values are reproduced from the source meta-analyses.

Evidence Class	Risk Factor	Effect Estimate (95% CI)	*n* Studies	Total N	*p*-Value	I^2^ (%)	Egger *p*
Class I (Convincing)	Short menstrual cycle (<27 days)	OR 1.68 (1.48–1.89)	18	42,318	4.2 × 10^−12^	31	0.41
Class II (Highly suggestive)	Early menarche (<11–12 years)	OR 1.40 (1.25–1.57)	12	28,471	3.8 × 10^−9^	58	0.22
Class II	Low BMI/underweight	OR 0.80 (0.72–0.89) [protective effect of obesity]	15	35,204	2.1 × 10^−7^	64	0.38
Class III (Suggestive)	Dioxins/PCB exposure	OR 1.30 (1.12–1.51)	9	21,118	8.4 × 10^−4^	48	0.31
Class III	Family history of endometriosis	OR 1.95 (1.57–2.41)	11	25,762	3.2 × 10^−4^	72	0.18
Class III	Elevated IL-6, TNF-α (peritoneal fluid)	MD 8.3 pg/mL (6.1–10.5)	14	1427	4.7 × 10^−4^	85	0.09
Class IV (Weak)	Vegetable intake (protective)	RR 0.59 (0.45–0.77)	8	8213	2.3 × 10^−4^	78	0.04
Class IV	Caffeine > 300 mg/day	RR 1.30 (1.04–1.62)	5	6094	0.022	41	0.67
Class IV	Dairy products (protective)	RR 0.87 (0.79–0.96)	7	9841	0.005	33	NR
NS	Cigarette smoking	RR 1.05 (0.92–1.19)	10	24,015	0.48	12	0.55
NS	OCP use (historical)	Confounded by reverse causality	16	38,229	NR	89	NR

Notes. Class assignment follows the adapted Fusar-Poli and Radua framework. The downgrading of “Vegetable intake” to Class IV despite *p* < 10^−3^ reflects significant small-study effects (Egger *p* = 0.04). Where Egger’s test is reported as NR, the source meta-analysis included fewer than 10 primary studies, in which case publication-bias testing has insufficient statistical power. BMI = body mass index; OCP = oral contraceptive pill; PCB = polychlorinated biphenyls; NR = not reported in source; CI = confidence interval; MD = mean difference; OR = odds ratio; RR = relative risk.

**Table 4:** Diagnostic performance and clinical readiness of non-invasive biomarker approaches, with validation cohort details [26,27,28,42]. AUC = area under the receiver operating characteristic curve; BDNF = brain-derived neurotrophic factor; CI = confidence interval; ML = machine learning; miRNA = microRNA; NR = not reported in source.

Biomarker/Test	*n* Studies	Total N (Validation)	Sensitivity	Specificity	AUC (95% CI)	Clinical Readiness
CA-125 (single marker)	18	3420	52–78%	77–95%	0.74 (0.69–0.79)	Clinically available (rule-in)
CA-125 + BDNF + clinical (Oxford IVD test)	1	79	46%	100%	NR	Initial validation only [28]
miRNA panels (serum, ML-derived)	7	582	≥90%	≥85%	0.91 (0.85–0.96)	Pre-clinical stage [27]
miRNA in adolescents (Yale 2025)	1	~50 (discovery cohort)	NR	NR	NR (859 differentially expressed miRNAs)	Discovery/research stage [26]
PromarkerEndo (10 plasma proteins)	2	704 (combined development + validation)	up to 98%	up to 96%	0.997 (0.989–1.000), stage IV	Clinical trials [28]
Endotest (salivary miRNA)	2	~200	95–100%	95–100%	0.94 (NR)	Limited single-center validation
AI + ultrasound/MRI (IMAGENDO)	1	~350	NR	NR	0.906 (0.872–0.940), vs. 0.65 conventional	Research stage [42]

Notes. The very high AUC values for some platforms (PromarkerEndo, IMAGENDO) derive from internal or single-center validation and should be interpreted with caution, as discussed in Section 3.5. Prospective multi-center validation in unselected clinical populations is required before clinical implementation.

**Table 5:** Comparative treatment effectiveness with evidence classification and full statistical parameters. Effect estimates reproduced from source meta-analyses. COC = combined oral contraceptive; GnRH = gonadotrophin-releasing hormone; IVF = in vitro fertilization; NA = not applicable (single RCT); NR = not reported; OR = odds ratio; RCT = randomized controlled trial; RR = relative risk.

Intervention	Comparator	Key Outcome	Effect (95% CI)	*n* Studies	Total N	*p*-Value	I^2^ (%)	Egger *p*	Evidence Class	Source
Dienogest 2 mg/day (post-op)	Expectant management	Recurrence (OR)	0.30 (0.18–0.53)	14 (RCTs)	2137	8.2 × 10^−7^	41	0.28	Class II	Zakhari 2021 [29]
GnRH agonist (post-op)	Expectant management	Recurrence (OR)	0.35 (0.22–0.56)	9 (RCTs)	1654	2.4 × 10^−7^	38	0.42	Class II	Zakhari 2021 [29]
Extended-cycle progestins	COC	Pain recurrence (OR)	0.78 (0.61–0.99)	1 (PRE-EMPT RCT, multicenter)	900	0.041	NA	NA	Class III ^1^	Cooper 2024 [31]
Cystectomy	Fenestration/ablation	Endometrioma recurrence (OR)	0.41 (0.24–0.71)	8	1243	1.2 × 10^−3^	47	0.36	Class III	Wattanayingcharoenchai 2021 [30]
Post-op hormones + IVF	IVF alone	Pregnancy rate (OR)	1.48 (1.18–1.86)	12	2851	6.4 × 10^−4^	52	0.41	Class III	Bourdon 2024 [32]
GnRH antagonist + add-back	GnRH agonist	Bone density preservation	SMD 0.42 (0.28–0.56)	6	1124	8.3 × 10^−4^	28	NR	Class III	Systematic review
Vegetable intake (dietary)	Low vegetable intake	Endometriosis risk (RR)	0.59 (0.45–0.77)	8	8213	2.3 × 10^−4^	78	0.04	Class IV	Neri 2025 [25]
Caffeine > 300 mg/day	Low caffeine intake	Endometriosis risk (RR)	1.30 (1.04–1.62)	5	6094	0.022	41	0.67	Class IV	Neri 2025 [25]

Notes. ^1^ The PRE-EMPT trial is a single multicenter RCT; consequently, I^2^ and Egger statistics are not applicable. Class III is assigned based on the strength of this high-quality RCT in lieu of pooled meta-analytic data.

## Data Availability

This article is a review and does not report original data. All information is derived from previously published studies cited in the reference list.

## Supplementary Materials

The following supporting information can be downloaded at https://www.mdpi.com/article/10.3390/jcm15124583/s1, Supplementary File S1—Completed PRIOR 2023 reporting checklist with page references; Supplementary File S2—Complete search strategies for PubMed, Embase, Cochrane Library, and Scopus, with dates of execution and number of records retrieved; Supplementary File S3—Primary-study overlap matrices and domain-specific CCA values; Supplementary Table S1—Full characterization of all 52 included systematic reviews and meta-analyses (16 columns × 52 rows); Supplementary Table S2—AMSTAR-2 16-domain quality matrix for all 52 reviews. Reference [44] is cited in the supplementary materials.
