# Supplementary material for: Epidemiology, Risk Factors, Diagnosis, and Comorbidities of Endometriosis: An Umbrella Review
Source: J Clin Med. 2026 Jun 12;15(12):4583. doi: 10.3390/jcm15124583 (PMC13302086; doi:10.3390/jcm15124583)
Supplement: Supplementary file 1 [file jcm-15-04583-s001.zip › Supplementary_File S1_PRIOR_2023_Checklist (1).pdf]

Supplementary File S1

PRIOR 2023 Reporting Checklist for Overviews of Reviews

Reference: Gates M, Gates A, Pieper D, Fernandes RM, Tricco AC, Moher D, et al. Reporting guideline for overviews of reviews of healthcare interventions: development of the PRIOR statement. *BMJ* 2022;378:e070849. doi:10.1136/bmj-2022-070849

Note. *Page numbers refer to the revised manuscript. Items reported in supplementary materials are indicated with the corresponding file name (S1, S2, S3). "NA" indicates items not applicable to this umbrella review.*

Item #	PRIOR Item description (paraphrased)	Page / Section in manuscript	Status
**TITLE**			
**1**	Identify the report as an overview of reviews in the title.	Title page ("An Umbrella Review of Systematic Reviews and Meta-Analyses")	✓ Reported
**ABSTRACT**			
**2**	Provide a structured abstract: background, objectives, eligibility criteria, information sources, methods (including risk of bias / quality appraisal), results (number of included reviews, key findings), and conclusions.	Abstract, p. 1–2	✓ Reported
**INTRODUCTION**			
**3**	Describe the rationale for the overview in the context of what is already known.	Introduction, p. 2	✓ Reported
**4**	Provide an explicit statement of the objective(s) or question(s) the overview addresses.	Introduction, end of section, p. 3 (four explicit objectives listed)	✓ Reported
**METHODS**			
**5**	Specify inclusion and exclusion criteria for reviews and how reviews were grouped for syntheses (PICO/PECO).	Methods §2.2 (Eligibility), p. 3–4	✓ Reported
**5a**	Describe whether and how primary studies within reviews were considered.	Methods §2.6 (CCA / overlap), p. 5	✓ Reported
**6**	Specify all databases, registries, and other sources searched; specify the date when each source was last searched.	Methods §2.3, p. 4 — and Supplementary File S1	✓ Reported
**7**	Present the full search strategies for all databases (provide as supplement).	Methods §2.3 (PubMed string shown); full strategies in Supplementary File S1	✓ Reported

Item #	PRIOR Item description (paraphrased)	Page / Section in manuscript	Status
**8**	Specify the methods used to decide whether a review met the inclusion criteria, including how many reviewers screened and any automation tools used.	Methods §2.4 (duplicate independent screening, κ values), p. 4–5	✓ Reported
**9**	Specify the methods used to collect data from included reviews (e.g., piloted forms, independent extractors).	Methods §2.4, p. 4–5	✓ Reported
**10**	List and define all variables for which data were sought.	Methods §2.4, p. 4–5 (authors, year, n studies, total N, effect estimates, I^2, publication bias, AMSTAR-2)	✓ Reported
**11**	Describe methods for handling and presenting characteristics of primary studies within included reviews.	Methods §2.6 (CCA); Results §3.1, Supplementary Table S1	✓ Reported
**12**	Specify the methods used to assess methodological quality of included reviews (e.g., AMSTAR-2, ROBIS).	Methods §2.5 (AMSTAR-2 with 7 critical domains), p. 5	✓ Reported
**13**	Specify methods to handle overlap of primary studies across included reviews.	Methods §2.6 (CCA method, citation matrices); Supplementary File S2	✓ Reported
**14**	Describe synthesis methods including how data were summarized (narrative, tabular, quantitative).	Methods §2.7 (Fusar-Poli & Radua scheme); Results §3.2–§3.7	✓ Reported
**15**	Describe methods for assessing certainty of the body of evidence (e.g., GRADE, Fusar-Poli & Radua).	Methods §2.7; Table 1 (evidence classification scheme), p. 6	✓ Reported
**16**	Describe any methods used to assess risk of bias in primary studies (as reported by included reviews).	Methods §2.4 (extracted from included reviews); discussed in Results	✓ Reported
**17**	Describe any methods used to assess risk of reporting bias and publication bias.	Methods §2.4 (Egger test results extracted where reported); Supplementary Table S1	✓ Reported
**18**	Describe any subgroup analyses, sensitivity analyses, or meta-regression performed.	NA (this umbrella review did not re-pool	✓ Reported

Item #	PRIOR Item description (paraphrased)	Page / Section in manuscript	Status
		primary data; subgroup analyses are those reported in the included meta-analyses)	
**RESULTS**			
**19**	Provide the numbers of records identified, screened, assessed for eligibility, and included, ideally with a PRISMA-OvR flow diagram.	Results §3.1, p. 7; Figure 1 (PRISMA-OvR flow diagram)	✓ Reported
**20**	Cite each included review and present its characteristics.	Results §3.1, Table 2 (thematic summary); full details in Supplementary Table S1	✓ Reported
**21**	Present overlap of primary studies across included reviews (CCA or matrix).	Results §3.1.1; Supplementary File S2 (overlap matrices and CCA values per domain)	✓ Reported
**22**	Present results of methodological quality assessment (AMSTAR-2) for each included review.	Results §3.1, Figure 2 (representative subset); Supplementary Table S2 (all 52 reviews)	✓ Reported
**23**	Present results of certainty of evidence assessment for each association/outcome.	Results §3.3, §3.5, §3.6 (evidence classes I–IV/NS in Tables 3, 4, 5)	✓ Reported
**24**	Present the synthesized findings (descriptive, tabular, or quantitative) for each clinical domain/outcome.	Results §3.2–§3.7, Tables 2–5, Figures 3–6	✓ Reported
**25**	Present results of any sensitivity or subgroup analyses (if performed).	NA — see Item 18	✓ Reported
**DISCUSSION**			
**26**	Provide a general interpretation of the results in the context of other evidence; discuss limitations of included reviews and the overview itself.	Discussion §4, p. 14–16 (Strengths and Limitations subsection)	✓ Reported
**26a**	Discuss implications of the results for practice, policy, and future research.	Discussion §4 (clinical implications); Conclusions §5 (research priorities)	✓ Reported

Item #	PRIOR Item description (paraphrased)	Page / Section in manuscript	Status
**OTHER INFORMATION**			
**27**	Provide registration information for the overview, including registry name and registration number (or state if not registered).	Methods §2.1 (PROSPERO CRD420261378862), p. 3	✓ Reported
**27a**	Indicate where the overview protocol can be accessed, or state that a protocol was not prepared.	Methods §2.1 (protocol on PROSPERO); Author Contributions and Data Availability sections	✓ Reported
**27b**	Describe and explain any amendments to information provided at registration or in the protocol.	NA — no amendments made to the registered protocol	✓ Reported
**27c**	Describe sources of financial or non-financial support for the overview and the role of funders.	Funding section, p. 17 (Ministry of Healthcare RK, BR27308739)	✓ Reported
**27d**	Declare any competing interests of overview authors.	Conflicts of Interest section, p. 17	✓ Reported
**27e**	Report which of the following are available, where they can be found, and under which conditions they may be accessed: template data collection forms; data collected from included systematic reviews; analytic code; any other materials used.	Data Availability Statement, p. 17; Supplementary Files S1–S3	✓ Reported

Completion summary

Total PRIOR 2023 items: 27 main items + 19 sub-items. Items reported in the manuscript: 25 main items + 17 sub-items. Items marked NA (justified): 2 (items 18 and 25 — sensitivity/subgroup analyses were not re-performed at the umbrella-review level, as this overview synthesizes findings from previously published meta-analyses rather than re-pooling primary data; the relevant analyses are reported within the included reviews themselves and are referenced in Supplementary Table S1).

Statement of compliance

This umbrella review is reported in accordance with the PRIOR 2023 (Preferred Reporting Items for Overviews of Reviews) statement [Gates et al., BMJ 2022;378:e070849]. The protocol was prospectively registered in PROSPERO (CRD420261378862) prior to the commencement of formal screening.
