# Supplementary material for: Epidemiology, Risk Factors, Diagnosis, and Comorbidities of Endometriosis: An Umbrella Review
Source: J Clin Med. 2026 Jun 12;15(12):4583. doi: 10.3390/jcm15124583 (PMC13302086; doi:10.3390/jcm15124583)
Supplement: Supplementary file 1 [file jcm-15-04583-s001.zip › Supplementary_File S2_Search_Strategies.pdf]

Supplementary File S2

Complete Search Strategies for All Four Electronic Databases

This file provides the complete, reproducible search strategies executed in PubMed, Embase, Cochrane Library, and Scopus on 15 March 2026, in accordance with PRIOR 2023 and PRISMA-S 2021 reporting standards. Search strategies were developed in consultation with a medical librarian. For each database, the exact search string, dates of execution, applied filters, and the number of records retrieved are reported.

Language was restricted to English and Russian. Document types were limited to systematic reviews, meta-analyses, and overviews of reviews. The search was last updated on 15 March 2026.

Summary of records retrieved

Database	Records retrieved	Date of search	Filters applied	Search platform
**PubMed (MEDLINE)**	1,847	15 March 2026	English, Russian; SR, MA, overview	https://pubmed.ncbi.nlm.nih.gov
**Embase**	2,103	15 March 2026	English, Russian; SR, MA, overview	Elsevier Embase.com
**Cochrane Library (CDSR + CENTRAL)**	412	15 March 2026	English, Russian; CDSR; SR/MA in CENTRAL	Cochrane Library (Wiley)
**Scopus**	1,291	15 March 2026	English, Russian; document type filters	Elsevier Scopus
**Manual searching (reference lists + PROSPERO registry)**	47	16–20 March 2026	Manual screening	Reference lists of included reviews; PROSPERO
**TOTAL (before deduplication)**	**5,700**	—	—	—

1. PubMed (MEDLINE) — full search string

Search platform: https://pubmed.ncbi.nlm.nih.gov | Date executed: 15 March 2026 | Records retrieved: 1,847

```
(("Endometriosis"[MeSH Terms]    OR "endometriosis"[Title/Abstract]    OR
"endometrioma"[Title/Abstract]    OR "deep infiltrating endometriosis"[Title/Abstract]    OR
"adenomyosis"[Title/Abstract])  AND  ("Systematic Review"[Publication Type]    OR "Meta-
Analysis"[Publication Type]    OR "systematic review"[Title/Abstract]    OR "meta-
analysis"[Title/Abstract]    OR "meta analysis"[Title/Abstract]    OR "overview of
reviews"[Title/Abstract]    OR "umbrella review"[Title/Abstract]    OR "review of
reviews"[Title/Abstract])) AND ("2016/01/01"[PDAT] : "2026/03/31"[PDAT]) AND (English[Language]
OR Russian[Language])
```

Filters applied: Date of publication 2016/01/01–2026/03/31; Languages: English, Russian; Article types: Systematic Review, Meta-Analysis.

2. Embase — full search string

Search platform: Elsevier Embase.com (Emtree mapping enabled) | Date executed: 15 March 2026 | Records retrieved: 2,103

```
('endometriosis'/exp     OR 'endometrioma'/exp     OR 'deep infiltrating endometriosis':ti,ab
OR 'adenomyosis'/exp) AND ('systematic review'/exp     OR 'meta analysis'/exp     OR 'systematic
review':ti,ab     OR 'meta-analysis':ti,ab     OR 'umbrella review':ti,ab     OR 'overview of
reviews':ti,ab) AND [2016-2026]/py AND ([english]/lim OR [russian]/lim) AND ('article'/it OR
'review'/it)
```

Filters applied: Publication year 2016–2026; Languages: English, Russian; Article types: Article, Review.

3. Cochrane Library — full search string

Search platform: Cochrane Library (Wiley); searched Cochrane Database of Systematic Reviews (CDSR) and CENTRAL | Date executed: 15 March 2026 | Records retrieved: 412

```
#1   MeSH descriptor: [Endometriosis] explode all trees #2   (endometriosis OR endometrioma OR
"deep infiltrating endometriosis" OR adenomyosis):ti,ab,kw #3   #1 OR #2 #4   ("systematic
review" OR "meta-analysis" OR "umbrella review" OR "overview of reviews"):ti,ab,kw #5   #3 AND
#4 in Cochrane Reviews and Trials #6   #5 with Publication Year from 2016 to 2026
```

Filters applied: Publication year 2016–2026; Languages: English, Russian.

4. Scopus — full search string

Search platform: Elsevier Scopus | Date executed: 15 March 2026 | Records retrieved: 1,291

```
( TITLE-ABS-KEY ( endometriosis OR endometrioma                    OR "deep infiltrating
endometriosis"                   OR adenomyosis ) AND TITLE-ABS-KEY ( "systematic review" OR
"meta-analysis"                    OR "meta analysis"                     OR "umbrella review"
OR "overview of reviews" ) ) AND PUBYEAR > 2015 AND PUBYEAR < 2027 AND ( LIMIT-TO
( LANGUAGE,"English" )       OR LIMIT-TO ( LANGUAGE,"Russian" ) ) AND ( LIMIT-TO
( DOCTYPE,"re" )       OR LIMIT-TO ( DOCTYPE,"ar" ) )
```

Filters applied: Publication year 2016–2026; Languages: English, Russian; Document types: Review (re), Article (ar).

5. Manual searching

In addition to the electronic database searches, manual searches were performed by two reviewers independently between 16 and 20 March 2026. Sources included:

• Reference lists of all 52 finally included systematic reviews and meta-analyses (citation chaining);

• The PROSPERO international prospective register of systematic reviews (https://www.crd.york.ac.uk/prospero/), searched using the terms "endometriosis" AND ("umbrella" OR "overview" OR "systematic");

• Recent conference proceedings of the World Congress on Endometriosis (2023, 2025) and ESHRE Annual Meeting (2023–2025).

These manual searches retrieved 47 additional records, of which 35 were unique after deduplication against the electronic database results.

6. Deduplication

All retrieved records were imported into EndNote 21 and subsequently into the Rayyan systematic review platform (https://www.rayyan.ai/) for deduplication and screening. Deduplication was performed in two stages:

Stage 1 — automated deduplication of database records: 1,435 duplicates were identified and removed from the combined 5,653 database records, leaving 4,218 unique records.

Stage 2 — manual cross-checking of the 47 manual-search records against the database records: 12 records were found to be duplicates of records already retrieved by the databases and were removed, leaving 35 unique records from manual searching.

The final pool of unique records for title/abstract screening was therefore 4,218 + 35 = 4,253 records, as reflected in the PRISMA-OvR flow diagram (Figure 1).

Notes

All search strategies, including the iterative pilot searches that informed the final strategies, are available from the corresponding author on reasonable request. The complete EndNote (.enl) and Rayyan project files, including the full list of excluded records with reasons, are available as restricted-access supplementary materials and can be obtained via the corresponding author.
